# Do golden snub-nosed monkeys use deceptive alarm calls during competition for food?

**DOI:** 10.1016/j.isci.2023.106098

**Published:** 2023-02-02

**Authors:** Tiantian Wang, Yuchen Kong, He Zhang, Yuhang Li, Rong Hou, Derek W. Dunn, Xiduo Hou, Kang Huang, Baoguo Li

**Affiliations:** 1 Shaanxi Key Laboratory for Animal Conservation, College of Life Sciences, Northwest University, Xi’an 710069, China; 2 Qingyang No.6 Middle School, Qingyang 745000, China

**Keywords:** Biological sciences, Zoology, Ethology

## Abstract

Tactical deception can be beneficial for social animals during intra-specific competition. However, the use of tactical deception in wild mammals is predicted to be rare. We tested whether a food-provisioned free-ranging band of golden snub-nosed monkeys (*Rhinopithecus roxellana*) use alarm calls in a functionally deceptive manner to gain access to food resources, whether the rate of deceptive alarm calls varies among individuals, and whether there are any counter-deception behaviors. We used a hexagonal camera array consisting of 10 cameras to record videos during feeding, which allowed us to identify individual alarm callers. We found evidence that these monkeys use deceptive alarms and that adult females were more likely to use such calls than other individuals. The monkeys increased their rates of response to alarm calls when competition for food was high. However, we found no direct evidence of any counter-deception strategies.

## Highlights

• Golden snub-nosed monkeys use deceptive alarms during competition for food
• Females utter alarm calls more frequently than other individuals
• Monkeys increase their response rates when competition for food is high
• No direct evidence of counter-deception was found

## Graphical abstract

**Figure:**
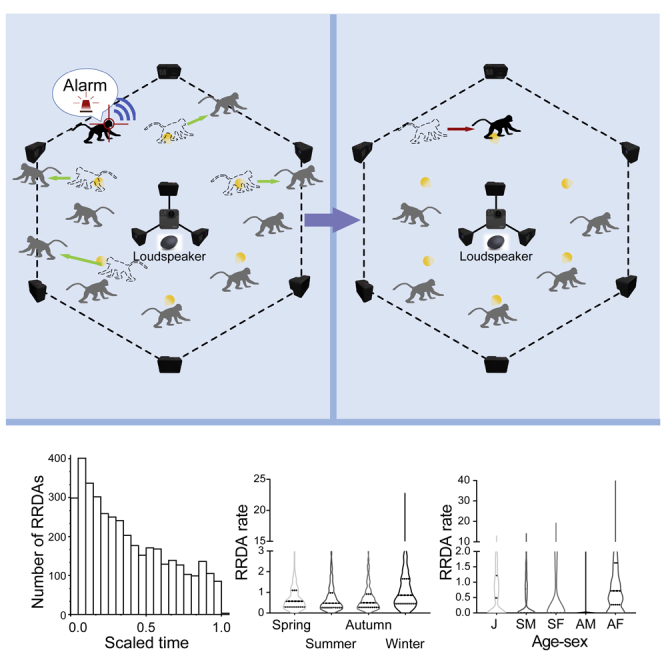

## Introduction

Many animals live in groups, which has many benefits such as reduction in predation risk, an increase in reproductive opportunities, ease of information transfer, better access to food (e.g., shared foraging and assistance in hunting), and the spread of cultural traits via learning. However, living in groups also has costs, such as an increased risk of disease transmission, a higher risk of inbreeding, and increased competition for resources.^1^^,^^2^^,^^3^ Sociality has thus enabled a diverse range of cooperative and competitive behavioral strategies between conspecifics to have evolved.

With the evolution of cooperative and competitive behavioral strategies in animals, deceptive behaviors emerged as a form of competition avoidance.^4^ Social animals may benefit from deceptive behaviors with the most widely used definition of deception being “tactical deception” also known as “functional deception”.^5^^,^^6^^,^^7^ Functional deception in communicative behaviors consists of three important criteria: (i) functional deception acts as part of the normal repertoire of the sender, (ii) it is emitted out of context, such that the receiver is likely to misinterpret what the sender signifies, and (iii) the receiver’s typical response benefits the sender.^8^^,^^9^ When deceptive signaling is common, there should be selection for individuals to anticipate such behaviors. Counter-deception is defined as a behavior, not necessarily deceptive itself, which functions to reduce the success of another’s deceptive behavior.^10^ By particular contexts, callers or certain structural signals, the receivers can be expected to make different decisions in how to respond to a signal based on the costs and benefits of certain responses, to avoid the costs of being deceived.^10^^,^^11^

Alarm calls are commonly used by birds and mammals^12^ and are commonly uttered in the presence of potential danger, e.g., when a predator is close.^13^ The information transmitted by alarm calls enables other individuals to take corresponding measures to avoid risks, thus improving the survival rate of individuals or groups.^14^ Alarm calls have many benefits when deployed in groups, such as the “many-eyes effect” and cooperative defense.^15^^,^^16^ However, calling will potentially expose callers to increased danger. Therefore, explaining the benefits of alarm calls to callers can be challenging (Hauser, 1996). Several hypotheses have been proposed, such as those based on kin selection and individual selection.^17^^,^^18^^,^^19^^,^^20^

Deceptive alarm calls are the most common form of functional deception involving vocalizations.^21^ For example, male barn swallows (*Hirundo rustica*) make deceptive alarm calls during the period of female egg-laying as a mechanism to ensure offspring paternity.^22^ Great tits (*Parus major*) are more likely to give alarm calls as competition for food increases.^23^ Fork-tailed drongos (*Dicrurus adsimilis*) use deceptive alarm calls to mimic other species in order to steal food,^24^ with similar behaviors recorded in both foxes and primates. Although no danger is apparent, Arctic foxes (*Alopex lagopus*) make warning calls to drive away cubs to regain food items.^25^^,^^26^ Tufted capuchin monkeys (*Cebus apella nigritus*) use alarm calls to distract other individuals during competitive situations, alleviating some of the costs associated with contest competition for food.^25^^,^^26^

We examined the use of “chuck” calls during competition for food^27^ in a wild-breeding band of golden snub-nosed monkeys (*Rhinopithecus roxellana*, hereafter abbreviated as GSMs) who live in a multi-level society with the *one-male multifemale units* (OMUs) as the main reproductive units.^28^ The “chuck” call is emitted as a response to a variety of environmental cues, such as the immediate presence of predators or humans, sudden sounds of breaking or falling objects, and other various disturbances, and as an alert/alarm signal.^27^ Upon hearing chucks, individuals may elicit anti-predator behaviors such as by making their own chuck calls, climbing up trees,^27^ and exhibiting overt vigilance. We use alarm calls to refer to the “chuck” calls in the following text.

According to Wheeler,^21^ an alarm call produced in the experimental feeding contexts was considered a *resource-related* (functionally) *deceptive alarm* (RRDA) call if certain criteria were met that eliminated other likely explanations for call production. We aimed to identify any RRDAs in GSMs, and if present, to record the patterns of RRDA deployment and to determine if there are also any counter-deception behaviors. We focused on three predictions based on the premise that anti-predator calls are used deceptively during feeding to enable some individuals to usurp others to gain access to food.

Prediction 1: if deceptive alarm calls are used by GSMs, the rate of alarm calls will be positively correlated with the intensity of food competition. Because the intensity of food competition cannot be directly measured, we used alternative variables likely correlated with food availability. For instance, natural food items are especially limited during winter,^29^ so we used “season” as one predictor variable. Food provisioning was also conducted in the morning and afternoon, so the time interval between food availability was longer in the morning than in the afternoon. Energy demands may also increase during adverse weather conditions. Each of these factors may thus result in individual monkeys being hungrier, therefore intensifying competition for food.^30^ Similarly, average feeding times across the OMUs present, the average rank of the OMUs present, and the number of OMUs present also likely reflect variation in the intensity of food competition. We therefore predict that RRDA rates will be highest during winter, during adverse weather conditions, in the morning, when average feeding times across all OMUs present are shorter, when the average rank of all OMUs present is high, when the number of OMUs present is high, and when feeding has been initiated.

Prediction 2: assuming Prediction 1 holds, if deceptive alarm calling varies among individuals, then age-sex class and social rank will predict the rate of alarm calls. Because dominants can easily take resources from subordinates through displacements, subordinates are expected to utter deceptive alarm calls more frequently.^21^^,^^23^ More specifically, we predict that subordinate individuals, adult females, and juveniles in low-ranked OMUs make alarm calls at high rates than other individuals.

Prediction 3: assuming Predictions 1 and 2 both hold, if counter-deception strategies are used, GSMs will be more likely to respond to the most reliable alarm callers and/or when competition is low. We predict that an alarm call is less likely to result in other monkey escaping or eliciting other responses when the caller is less reliable (adult female or juvenile, in a lower-ranked OMU, and faces toward or is inside the feeding area) or when the competition for food is high (e.g., during adverse weather, during winter, in the morning, when the duration of the feeding bout is short, at the beginning of a feeding bout, when the number of OMUs present is high, or when the time interval from the previous RRDA is long).

## Results

### OMU ranking

We recorded 1,039 agonistic interactions among the resident males (the adult males in OMUs) of different OMUs. The dominance proportions between OMUs and the dominance statistics are shown in Tables S1–S4.

### Descriptive statistics

We recorded 200 feeding bouts (50 feeding bouts for each season, where a feeding bout is a feed of the whole breeding band, begins when the first OMU enters the feeding site and ends when there are only two OMUs left in the feeding site) and obtained 25.3 TiB of raw and 125.49 h of video data. A total of 8,084 alarm calls were recorded during the study period, with 283 excluded due to being uttered by infants and 29 excluded due to being made before the feeding bout began. For the remaining 7,772 alarm calls, 221 were invoked by potential threats, 142 were invoked by conspecific aggression, 3,494 were responses to other alarms, and 3,915 were RRDAs.

**Figure 1:**
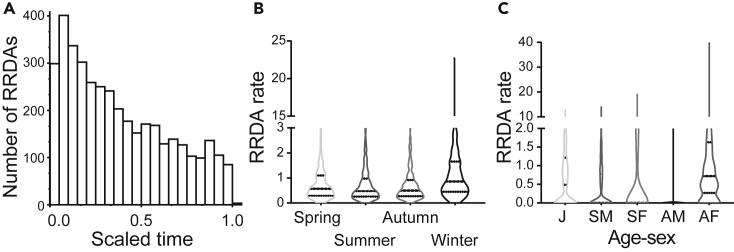
Comparisons of RRDA rates (A) Histogram of scaled times of RRDAs. The violin plot of RRDA rate (#RRDAs per individual per hour) in different seasons (B) and in different age-sex classes (C) with the dashed lines denoting quartiles.

The histogram of times of RRDA of each OMU during each feeding case (a feed of an OMU, which begins at the beginning of the feeding bout and ends when the last member of this OMU leaves the feeding site) and the violin plot of RRDA rate in different seasons are shown in Figure 1. The RRDA rate peaked at the beginning of the feeding case and then declined steadily over time. The overall RRDA rate was 0.792 ± 0.013 (Mean ± SE) times per individual per hour. The order of RRDA rate in different seasons is winter (1.145 ± 0.031) > spring (0.711 ± 0.026) > autumn (0.680 ± 0.022) > summer (0.633 ± 0.023).

### Predictions 1 & 2

#### RRDA rate

We extracted 9,485 individual feeding records from the dataset (File S4). Temperature showed high multi-collinearity with other variables and was thus excluded from the Poisson generalized linear mixed model (GLMM) (Table S5). The effects of the explanatory variables and subsequent analysis of deviance are shown in Tables 1 and S6, respectively. All factors significantly affected the RRDA rate, with the exception of OMU rank and OMU size. The RRDA rate was higher in the morning and during windy, foggy, or rainy weather and increased as the number of OMUs present also increased but decreased as the average feeding time across OMUs increased. Independent of all other explanatory variables in the model, the order of effects for each of the four seasons on RRDA rate is winter > autumn > spring > summer (ΔDeviance = 161.28, false discovery rate [FDR]-*Q* < 0.001). Multiple comparisons show significant differences between all season pairs except for spring-summer (Table S7). Females give RRDAs more frequently, and the order of effects of different age-sex classes on RRDA rate are SF (sub-adult females) > AF (adult females) > J (juveniles) > SM (sub-adult males) > AM (adult males) (ΔDeviance = 89.06, FDR-*Q* < 0.001, Table S8).

**Table 1:** Analysis of deviance for factors of RRDA rate

Factor	Δ Deviance	d.f.	P-value	FDR-Q
Season	161.276	3	**<0.001**	**<0.001**
TimeOfDay	9.242	1	**0.002**	**0.004**
#OMUs	18.323	1	**<0.001**	**<0.001**
Weather	6.137	1	**0.013**	**0.017**
AvgFeedTime	31.130	1	**<0.001**	**<0.001**
AvgRank	37.184	1	**<0.001**	**<0.001**
AgeSex	89.057	4	**<0.001**	**<0.001**
OMURank	3.246	1	0.072	0.081
OMUSize	0.751	1	0.386	0.386

*P*-value or FDR-Q below 0.05 were marked in bold.

#### Changes of RRDA rate over time

We extracted 1,042 feeding cases (File S5). RRDA intensity significantly decreased over time independent of other explanatory variables in the model (${\hat{\beta }}_{t}$ = −7.348 × 10^−4^, ΔDeviance = 785.53, *p* < 0.001). The same result was found by the Wilcoxon’s sign rank test (Z = 14.476, *p* < 0.001).

### Prediction 3

#### Escape rate

The escape rate is the proportion of alarm calls that result in any receivers to escape (climb up a tree or flee from the feeding site). Based on the extracted 3,915 RRDAs (File S6), the number of OMUs present (in the feeding site) and the average rank of the OMUs present were significantly correlated and were thus excluded from this analysis (Table S9). Logistic GLMM indicates that an RRDA with a longer time interval since the previous RRDA translates to a significantly higher escape rate ($\hat{\beta }$ = 1.305 × 10^−3^, ΔDeviance = 15.561, FDR-*Q* = 0.001, Tables 2 and S10). Season became insignificant after FDR adjustment (FDR-*Q* = 0.183), but all other variables did not significantly influence escape rate (Table 2).

**Table 2:** Analysis of deviance for factors of escape and response rates

	Factor	ΔDeviance	d.f.	p-value	FDR-*Q*
Escape rate	Direction	0.25	1	0.617	0.754
	Position	2.66	1	0.103	0.284
	OMURank	0.83	1	0.362	0.569
	AgeSex	8.69	4	0.069	0.254
	Season	8.72	3	**0.033**	0.183
	TimeOfDay	0.05	1	0.820	0.820
	Weather	0.10	1	0.747	0.820
	Duration	1.79	1	0.181	0.399
	Interval	14.73	1	**<0.001**	**0.001**
	ElapseTime	1.16	1	0.280	0.514
	Current#OMUs	0.42	1	0.518	0.712
Response rate	Direction	2.207	1	0.137	0.236
	Position	0.023	1	0.879	0.879
	OMURank	0.484	1	0.487	0.623
	AgeSex	11.446	4	**0.022**	0.053
	Season	29.568	3	**<0.001**	**<0.001**
	TimeOfDay	1.424	1	0.233	0.349
	Weather	6.048	1	**0.014**	**0.042**
	Duration	0.415	1	0.519	0.623
	AvgRank	9.177	1	**0.002**	**0.010**
	Interval	3.206	1	0.073	0.147
	ElapseTime	63.967	1	**<0.001**	**<0.001**
	Current#OMUs	0.129	1	0.719	0.785

*P*-value or FDR-Q below 0.05 were marked in bold.

#### Response rate

The response rate is the proportion of alarm calls that result in any response (including escapes and alarms). The number of OMUs present shows a high multi-collinearity and was thus excluded from this analysis (Table S9). Season, weather, average rank of the OMUs present (in the feeding site), and elapsed time (from the beginning of the feeding bout) significantly affected the response rate (Table 2). RRDAs are more likely to evoke a response in winter ($\hat{\beta }$ = 0.508), during adverse weather conditions ($\hat{\beta }$ = 0.184, ΔDeviance = 6.048, FDR-*Q* = 0.042), at the beginning of the feeding bout ($\hat{\beta }$ = −5.106 × 10^−4^, ΔDeviance = 63.967, FDR-*Q* < 0.001), and when the average rank of the OMUs present is low ($\hat{\beta }$ = 2.230 × 10^−1^, ΔDeviance = 9.177, FDR-*Q* = 0.010). The order of the response rate for different seasons is winter > autumn > summer > spring (ΔDeviance = 29.568, FDR-*Q* < 0.001, Tables S11 and S12). Age-sex class was non-significant after FDR adjustment (FDR-*Q* = 0.053), with all other variables not significantly affecting the response rate.

## Discussion

### Food competition and RRDA rate

We found that increased food competition resulted in significantly increased RRDA rate, which is consistent with a previous study of tufted capuchins (*C. apella nigritus*).^11^ The histogram of scaled times of RRDAs (Figure 1) indicates that the RRDA rate decreases as a function of elapsed time. These results concur with our Prediction 1 that the rate of alarm calls is positively correlated with the intensity of food competition, consistent with the use of deceptive alarms by GSMs.^7^

This suggests that RRDA is a strategy used during food competition in GSMs, and one individual will use an alarm call, causing others to flee, leaving the contested food behind. Such phenomenon has been observed many times, and we provide a video as an example: an adult female outside of the feeding site sends a deceptive alarm causing individuals in other OMUs to disperse, and then the caller enters the feeding site and feeds (File S8).

### Individual RRDA strategies

The social structure of GSMs is based on a dominance hierarchy.^31^ Individuals are also able to avoid some behaviors with high-energy demands such as aggressive behaviors.^32^ We found OMU rank did not affect the RRDA rate (Table 1), which failed to concur with our Prediction 2 and previous studies of other primate species, e.g., Wheeler.^21^

We speculate that this is due to the multi-level society of GSMs.^28^ The dominance rank in most other primate species is individual based, while that in GSMs is OMU based.^33^ The rank of an OMU is the likely result of the combined effects of all adult OMU members,^31^^,^^34^ while the dominance rank among females within the same OMU is unclear or inconsistent.^35^ We found that OMU rank did not significantly affect response and escape rates (Table 2). In other words, the RRDAs uttered by individuals from either high- or low-ranked OMUs have the same efficacy, so both high- and low-ranked OMUs can benefit from the same RRDAs. Individuals in high-ranked OMUs can also use RRDAs as a means to avoid costly inter-OMU conflicts.

The RRDA rates are significantly different among age-sex classes (Table 1, Figure 1), where the females (either adult or sub-adult) utter RRDAs more frequently than other age-sex classes. This does not fully align with our prediction 2 that subordinate individuals, adult females, and juveniles would make alarm calls at higher rates than other individuals. The difference in RRDA rates among age-sex classes can be explained by two alternatives: i) the social role hypothesis or ii) the food competition hypothesis.

The social role hypothesis is that females maintain long-term relationships with other females within the same OMU^36^ and thus make more social interactions than OMU-resident males and use RRDAs in their socializations with other females within the same OMU.

The food competition hypothesis is based upon the adult females being subordinate to adult males during food competition and thus uttering RRDAs more frequently. Resident males are dominant to other age-sex classes and may not need to use deceptive alarm calls to gain access to high-value food; juveniles take less food (they only take the embryo of maize) and are tolerated to a higher degree by other individuals (they play together and can freely enter the location where other OMUs are feeding).^37^

Fan et al. (2018) found that the rates of “chuck” calls are similar for males and females when the monkeys are not feeding, where 16 calls from 2 males and 223 calls from 16 females were recorded. Therefore, the difference in RRDA rates we found among different age-sex classes may be best explained by the food competition hypothesis.

### Existence of a counter-deception strategy

According to Wheeler and Hammerschmidt,^10^ interpretation of counter-deception is most straightforward if variation in responses is driven by structural differences between honest and deceptive alarm calls or by a decreased response rate to unreliable callers. We compared the acoustic structure for two versions of alarms (deceptive or environmental disturbance-elicited) sent by different adult males and found they have similar spectrograms and spectra (Figure S1). However, playback experiments and additional data are required to test statistically whether these two versions of alarms are the same and evoke similar responses in receiver individuals, respectively.

The RRDA rate of adult females is higher than that of other age-sex classes except for sub-adult females (Table S6). This suggests that a higher proportion of their alarm calls are deceptive if all individuals have the same probability of encountering danger and sending honest alarms. Although the alarm calls uttered by adult females and juveniles may be more unreliable, we found no significant difference in both escape and response rates among sex-age classes. The remaining caller-specific variables that may reflect the reliability of the alarm calls also cannot predict the escape and response rates. This fails to concur with our Prediction 3.

The response rate of alarm calls was higher in high-competitive contexts with a lower food supply but a higher food demand (e.g., during windy, foggy, or rainy weather, during winter, and at the beginning of a feeding bout, Table S11) than in low-competitive contexts. However, escape rates do not significantly differ except for the time interval from a previous alarm call (Table 2). These results are also inconsistent with those for tufted capuchins.^11^ These results are partially consistent with our Prediction P3. However, the correlation between contextual variables and the response of the receivers is not clear support for the counter-deception strategy because this can be explained by (i) the receivers ascribing different meanings to the alarm calls in different contexts (indicating counter-deception strategy) or by (ii) the receivers ascribing a similar meaning to the alarm calls in different contexts but making a contextual decision as to how to respond to the alarm calls given that meaning.^10^

### Summary

We found the rate of deceptive alarm calls increases when competition for food is high, suggesting that some GSMs may use deceptive alarm calls to gain access to food. We found that deceptive alarm strategies varied among individuals, with females uttering deceptive alarm calls more frequently than other individuals. The response rate to alarm calls was the highest when competition for food was high, while escape rate was only affected by the time interval from a previous alarm call. The evidence of counter-deceptive strategies in GSMs is insufficient.

### Limitations of the study

First, the alarm calls inside and outside the feeding site were respectively recorded by video playback and field observation, with the latter more likely to be missed by human observers. Second, we did not measure and compare the acoustic parameters (e.g., fundamental frequency and harmonic frequencies) of different versions of alarm calls (deceptive alarms and those that were environmental disturbance elicited). Third, playback experiments are needed to test whether deceptive alarms and environmental disturbance-elicited alarms have the same function. Fourth, the “chuck” may be used as a general alert or a distress call in a highly competitive context, rather than a deceptive alarm call.

## STAR★Methods

### Key resources table

**Table:** 

REAGENT or RESOURCE	SOURCE	IDENTIFIER
**Deposited data**		

Repository data	This paper	Mendeley Data: https://doi.org/10.17632/fthm2fnc24.1
All original code	This paper	Mendeley Data: https://doi.org/10.17632/fthm2fnc24.1

**Software and algorithms**		

MATLAB	www.mathworks.com	8.2.0.701
r	www.r-project.org	4.0.2
ffmpeg	www.ffmpeg.org	N-74286-ge5774f2
goldwave	www.goldwave.com	6.18
filmora	filmora.wondershare.com	9.0

**O****ther**		

Graphics card	Gigabyte Technology Inc.	GV-N105TOC-4 GL
Panoramic camera	GoPro Inc.	GoPro Fusion
Sport camera	GoPro Inc.	Hero 5 Black
Camera remote	GoPro Inc.	A857

### Resource availability

#### Lead contact

Further information and resource requests should be directed to the Lead Contact: Kang Huang (huangkang@nwu.edu.cn).

#### Materials availability

This study did not generate new unique reagents.

### Experimental model and subject details

#### Study site and subjects

The study was conducted in the *Dapingyu* (DPY) region of the Guanyinshan Nature Reserve, Qinling Mountains, Shaanxi province, central China (107°40ʹ-107°55ʹ E, 33°33ʹ-33°46ʹ N). The terrain is mountainous and the elevation of the reserve is 1150–2574 m above sea level.^38^ The climate is temperate with a long spring and winter, and a short summer and autumn.

GSMs are endemic to mountainous regions of central China. They exhibit a distinct male-biased sexual size dimorphism,^39^^,^^40^ and are mainly arboreal, primarily inhabiting the mid-to-lower canopy.^41^ GSMs have a complex multi-level social structure.^28^^,^^42^ A cohesive breeding band consists of several OMUs, each of which is made-up of a single adult male, several adult females, and their juvenile and infant offspring that tend to forage and socialize together.^33^ The breeding band is shadowed by an *all-male band* (AMB), which consists of bachelor adult males and sub-adult males excluded from the breeding band.^28^^,^^43^ There is a linear dominance hierarchy among OMUs,^31^ reflecting sometimes high competition for food within their groups.^33^^,^^44^^,^^45^^,^^46^ The OMUs of a breeding band maintain a close association and coordinate their activities. While each OMU is spatially and socially distinct, the individuals of the same OMU usually stay much closer to each other than to those of other OMUs, and most social interactions occur among the individuals within the same OMU.^36^^,^^47^ Vocalizations are important means of communication in GSMs,^27^ although detailed investigations in this species are still mainly at the descriptive stage.^27^

The study herd, DPY-herd, consisted of a breeding band of 6–7 OMUs (∼75 individuals) and one AMB (∼20 individuals). These monkeys have been provisioned food since 2010 to enable close observation and individual identification. We focused on adult males, adult females, sub-adults and juveniles in the breeding band, and the classification of age-sex class followed Zhang et al*.*^48^ During the study period (consisting of two intervals: Oct. 2020 to Jan. 2021 and Mar. 2021 to Jul. 2021), there were 66 individuals in the first interval (autumn and winter), comprising of 6 adult males, 29 adult females, 8 sub-adults and 22 juveniles, and 57 individuals in the second interval (spring and summer), comprising of 7 adult males, 28 adult females, 3 sub-adults and 19 juveniles. These individuals were identified based on facial and body characteristics.^31^

We selected six relatively flat sites as feeding sites, each 15 × 15 m, to enable us to exclude potential interference and to identify individuals. In the early morning, field guides led the monkeys to the feeding site where they were fed twice daily, first at about 10:00 a.m. and again at 3:00 p.m. We provided 10 kg of dry kernels of maize (*Zea mays*) for the entire breeding band each day (averaging 150 g per individual per day).^29^

Not all OMUs came to the feeding site, especially in summer. High-ranked OMUs have a high feeding priority, and the individuals in the same OMUs tend to feed and then leave the food provisioning site together. The provisioned foods do not meet the daily energy requirements of the entire breeding band and these monkeys still spent much time on consuming natural food.^33^

#### Ethical note

All research protocols reported here adhere to the regulatory requirements and were approved by the animal care committee of the Wildlife Protection Society of China (SL-2012-42). The research was under the supervision of Institutional Animal Care and Ethics Committee of Northwest University, China.

#### Aggressive behavior observation

We recorded the agonistic interactions between OMU adult males. These aggressive behaviors consisted of biting, fighting, chasing, threatening, supplanting and vocal threatening; submissive behaviors consisted of avoidance, fleeing and crouching, following the behavioral definitions of Qi et al*.*^43^ These data would be used to determine the dominance rank of each OMU.

#### Alarm behavior observation

Alarm behaviors were recorded via direct human monitoring (for alarms outside the feeding site), by video playback (for alarms inside the feeding site), and by the all-occurrence recording method. We placed 10 cameras around the feeding site to record behaviors during feeding, with one panoramic camera (GoPro Fusion, GoPro Inc.) placed in the center ∼3 m above the ground. Three additional cameras (Hero 5 Black, GoPro Inc.) were also fixed at the same point, each 1.5 m above the ground, with a further six Hero 5 Black cameras arranged around the feeding site and forming an equilateral hexagon with each side 7 m in length (Figure 2). A loudspeaker in the center was used to synchronize the clocks of all cameras, which allowed us to perform sound source location (section synchronization of camera clocks). The distance of an individual to the nearest camera was at most 4 m, which allowed us to identify the individuals from the video. The individual identifications from videos were validated against direct observations.

**Figure:**
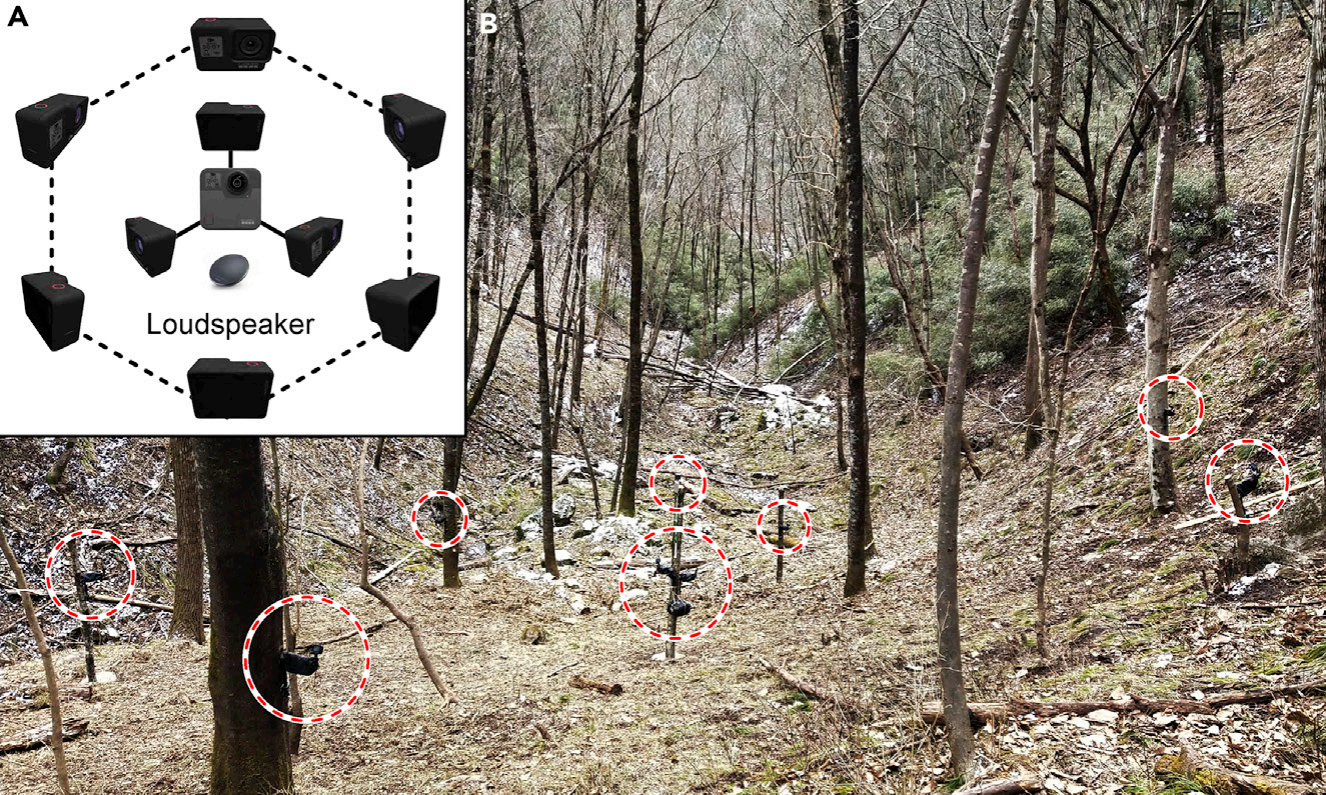
The arrange of feeding site and carema array (A) Schematic diagram of the hexagonal camera array and (B) photograph of the feeding site.

The RRDA rate is positively correlated with the intensity of food competition. In order to identify any RRDAs in GSMs, we indirectly estimated the intensity of food competition through other correlated indicators. During feeding, all of the members of the breeding band consume the provisioned food within 1 h of its distribution, and we define a feeding bout as a feeding duration of the whole breeding band. We recorded on video and the following data for each feeding bout: date, weather (clear, cloudy, windy, foggy or rainy), temperature, start time (when the first OMU entered the feeding site), stop time (when there were only two OMUs left in the feeding site), and both enter and exit times for each OMU.

The videos of each feeding bout recorded by the Hero 5 Black cameras were combined into a single multi-view video recording. We developed a MATLAB program (File S3) to mark the sound sources by crosshairs in the videos (sections sound source location and sound source mark up). This facilitated the identification of the individuals that made alarm calls.

To record the patterns of RRDA deployment and to identify any counter-deception behaviors, we recorded the following data for each alarm call (from either field observations or video analysis): time; caller; location (inside or outside the feeding site); distance to the feeding site for alarm calls uttered from outside the feeding area (categorized by < 2m, 2-5m, 5-10m and 10-20m); direction (facing toward or backwards the feeding site); type (defined as follows); responses of the other monkeys (additional alarm calls or escape behaviors) and the number of monkeys that left the feeding area. Alarm calls that resulted from human disturbances or were uttered by infants were excluded from further analyses.

We classified the alarm calls into four categories: (i) invoked by environmental or human disturbance (e.g., the immediate presence of predators or humans, sudden sounds of breaking or falling objects, other various disturbances); (ii) invoked by any aggression behaviors; (iii) response to other alarm calls (with an interval to previous alarm calls ≤ 5 s, section the cut-off of response alarm calls); and (iv) RRDA.^21^ The first two types of alarm call were not involved in further analyses, with the third type of alarm call used to calculate the response rate. The fourth type of alarm call was used to analyze the factors explaining variation in RRDA rate.

### Method details

#### Synchronization of camera clocks

We used a A857 Smart Remote (GoPro Inc.) to control all cameras, which can start and stop the recording of all cameras. However, there are still some differences in the start time. Besides, the clock frequencies of different cameras are slightly different (their frequencies differ by 1/48,000 to 3/48,000) and are changing during recording. This will interfere with the sound source location.

To solve this problem, we placed a loudspeaker in the center of the feeding site and played a synchronization audio to synchronize the clocks of cameras. In this audio, we made synchronization signals at an interval of 5 min. We also inserted a whistle sound with a duration of 0.5 s before the first and last synchronization signal. This is used to preliminarily locate the synchronization signal.

The waveform of the synchronization signal is shown in Figure 3A, lasts for 6.5 s and consists of 20 sine waves. The intervals between adjacent sine waves are randomly generated to prevent mismatch. Each sine wave lasts for 5 periods, and whose frequency ranges from 25 to 5000 Hz. Due to interference of both echo and obstacle, the wave received by the camera will last for more than 5 periods, and we could not precisely identify the offset of each camera. To prevent such interference, we inserted a 24,000 Hz high-frequency sine wave lasting for half periods in the crests of the first and last periods (Figure 3B). Then a high-frequency harmonic wave will yield a significant hollow in the wave received (Figure 3C), which can help us precisely synchronize the camera clocks. The errors can be reduced to 1/48,000 s.

**Figure:**
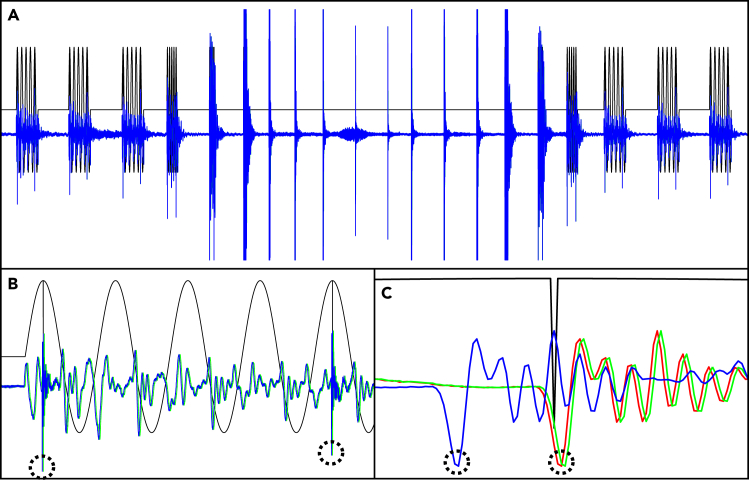
Synchronization of camera clocks (A) Alignment of waveforms of the synchronization signals. (B) The waveform of a sine wave that lasts for 5 periods. (C) The high-frequency harmonic wave at the crest of the first period. The black lines are the original signal, with the colored lines being the signals received by different channels. The significant hollows in the signals received are marked by circles.

By the method we describe above and with the coordinates of the cameras, we were able to calculate the time offset and frequency difference between parameters. We used camera #1 as the reference camera, and aligned and resampled the audio of the other cameras to the audio of camera #1.

For example, cameras #1 and #2 respectively received the same synchronization signal at 0 s and 0.1 s, and their distance to the loud speaker are 1 m and 7 m, respectively. Then we can obtain camera #2 is delayed for 0.1−6/340=0.082353 s. We removed such a duration of sound data in the beginning of camera #2 to align it with camera #1. If #2 is 0.082353 s ahead, we will pad a such a duration of sound to the beginning of #2.

For another example, cameras #1 and #2 respectively records 300 s and 300.0125 s between two synchronization signals. Assuming the sampling frequency of camera #1 is 48,000 Hz, then we can calculate the sampling frequency of camera #2 is 48,002 Hz. We resample the data of camera #2, i.e., delete two pulse points in the pulse-code modulation (PCM) format data every second, to ensure all cameras records the sounds in the same sampling frequency. If the sampling frequency camera #2 is lower than 48,000 Hz, e.g., 47,998 Hz, we will insert two pulse points every second.

#### Camera parameters determination

We used a total station to determine the coordinates of each camera (with an error of 0.01 m), and used plumb lines and surveying nails to help install the camera. These measures can help fix the coordinates of the cameras, but not the angles. Moreover, we installed and uninstalled the cameras every day, and it was hard to ensure that the positions and angles of these cameras were constant over time. Therefore, we used the images recorded by the cameras to determine these parameters.

We placed surveying nails in the wood pile or on trees, and each camera can record at least 4 reference objects of known coordinates (two cameras and two markers, Figure 4). These helped us adjust the camera angles during installation. These reference objects with known coordinates and their positions in the video can be measured, denoted by w. It is then possible to solve the camera parameters from w.

**Figure:**
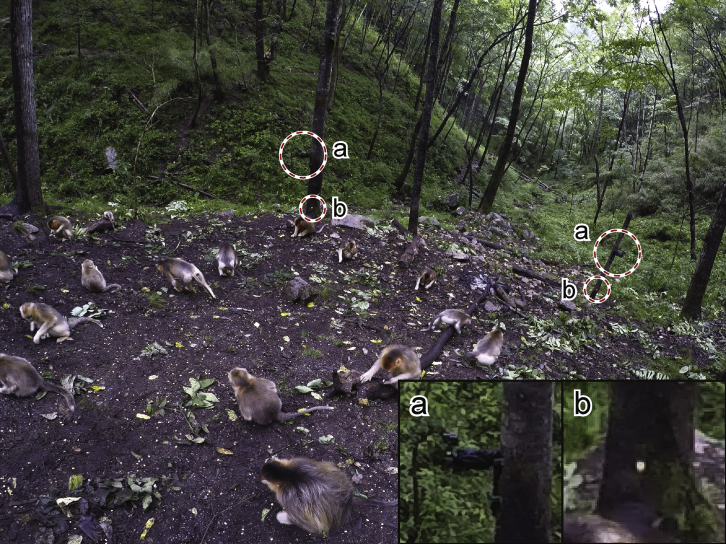
A photo of feeding site with the reference objects marked by the circles Partially enlarged are the camera (a) and the surveying nail (b).

We used six parameters to describe the coordinates and angles of a camera, including the coordinates (x,y,z) and three angles: the azimuth angle α, the elevation angle β and the polarization angle γ. These parameters are arranged in a vector θ=[xyzαβγ]. We can then calculate the predicted position $\hat{w}$ of a known reference object, which is a function of θ. By adjusting the camera parameters θ, we can make $\hat{w}$ been close to w. We use the Sum of Squared Errors (SSE) to evaluate the accuracy of θ:

$$SSE\left(\theta \right)=\sum_{i}{\left\|w_{i}-{\hat{w}}_{i}\right\|}_{2}^{2}\text{,}$$

and θ is optimized with the Newton’s method:

$$\hat{\theta }=\underset{\theta }{\mathrm{argmin}}SSE\left(\theta \right)\text{.}$$

However, a camera itself is also a reference object of the other cameras, and its coordinates are changing during calculation, we iteratively update θ for all cameras until their parameters are converged.

#### Sound source location

Assuming the audios are already synchronized, we are able to locate the sound source by the times of the same sound received by different cameras. That is, if the sound source is closer to a camera, then this camera will receive this sound earlier.

We used a predefined loudness threshold to activate the source sound location function, and used the cross-correlation coefficient (MATLAB function xcorr) to obtain the difference between the receiving times of different cameras (still using camera #1 as the reference). Let x be the coordinates of the sound source, $t_{i}$ be the receiving time of camera i, t be the sending time and $c_{i}$ be the coordinates of camera i. Then we can establish the following equations set:

$$\left\{ \begin{array}{c} {\left\|x-c_{1}\right\|}_{2}=v_{s}\left(t_{1}-t\right)\text{,}\\ {\left\|x-c_{2}\right\|}_{2}=v_{s}\left(t_{2}-t\right)\text{,}\\ \vdots \\ {\left\|x-c_{10}\right\|}_{2}=v_{s}\left(t_{10}-t\right)\text{.} \end{array}\right.$$

Where the left-hand side denotes the distance of the sound source to different cameras calculated from coordinates, and the right-hand side denotes the that calculated from time differences. The variable $v_{s}$ is the sound speed and is calculated from the current temperature and humidity

$$v_{s}=\sqrt{401.939\times \left(273.15+T\right)}\;m/s\text{,}$$

where T is the temperature in Celsius degree.

The above equation set is overdetermined, and can be solved with four cameras. Some cameras may fail to record the sound due to the far distance, and they will be excluded from calculation by the inconsistent cross-correlation coefficient. We usually used 5–6 cameras (including 4 in the center and 1–2 on the vertices of the hexagon).

The unknown variables are arranged into θ=[x,t], and the SSE is given by

$$SSE\left(\theta \right)=\sum_{i}{\left[{\left\|x-c_{i}\right\|}_{2}-v_{s}\left(t_{i}-t\right)\right]}^{2}\text{,}$$

The unknowns are still solved by the Newton’s method.

$$\hat{\theta }=\underset{\theta }{\mathrm{argmin}}SSE\left(\theta \right)\text{.}$$

#### Sound source mark up

We used the center of the camera as the origin and the view direction as the Y-axis, the up and right directions in the view plane as the Z-axis and X-axis, respectively, and then established a camera coordinate system. These can be calculated from the camera parameters.

As shown in Figure 5, let A be the sound source and $A^{\prime }$ be its projection in plane XOZ, we then calculated the angle α between the vector OA and Y-axis, and the angle θ between $OA^{\prime }$ and X-axis. Angle α was converted into the distance to image center r and yields the polar image coordinate (r,θ) of the sound source, which was then converted into the pixel coordinate (u,v).

**Figure:**
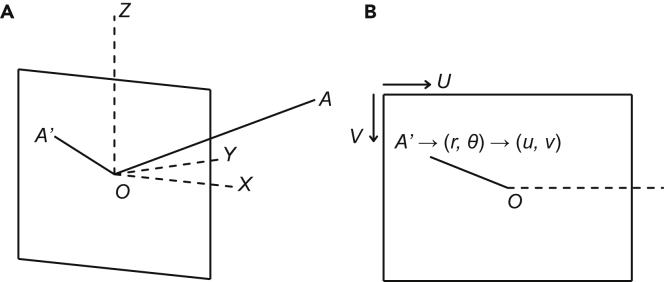
An illustration of coordination transformation (A) the camera coordinate system and (B) the polar image coordinate and pixel coordinate systems.

Next, we assumed the sound source is a sphere with a radius of 0.2 m, and calculated the radius of the sphere projection in image (in pixels) using the same method. We drew a crosshair using the calculated positions and radius using AAS subtitle to mark the sound source. During replay, we filtered the alarm calls by human ear, identified the sender, and recorded the information.

#### Video and audio processing

**Merge panoramic videos:** The panoramic videos are merged into a 4K 30 FPS video file (in H264 format) by GoPro Fusion Studio v1.3 (GoPro Inc.).

**Combine videos**: To be compatible with the FAT32 file system, a continuous video is saved in multiple files. We used ffmpeg to combine the videos of each camera for each feed into a single file.

**Extract audios**: The original PCM formatted WAV audio files have four channels, the left and the right channels were extracted by ffmpeg and written into a single WAV file.

**Transcoding**: We used GigaByte GV-N105TOC-4 GL graphics card to encode the video stream into H265 format with the hevc_nvenc encoder by ffmpeg. The audio stream was not processed.

**Snapshots**: We took eight snapshots (one for each minute) from the video for each camera (Figure 6). These were used to measure the positions of the reference objects (Figure 4).

**Figure:**
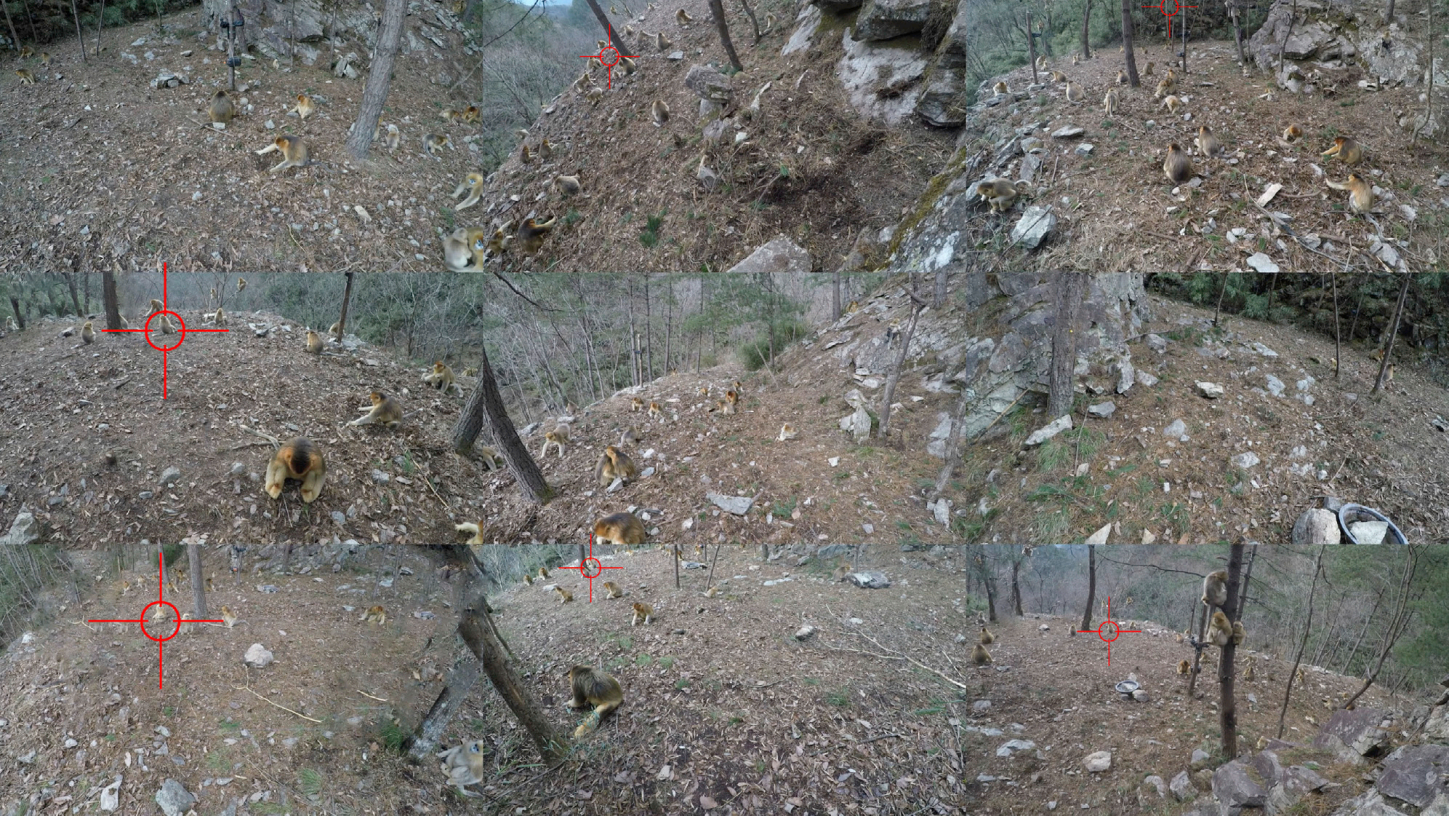
An illustration of sound source localization and mark up in the combined multi-view video

**Measure:** We used a picture processing software to measure the positions of all reference objects (cameras and surveying nails) and the loud speaker (in pixels). These positions are written into config.txt.

**Run****MATLAB****function step1()**: this consists of the camera parameters determination, first and last synchronization signals identifications, plotting waveforms around the high-frequency sine waves (to help obtain the time offset, Figure 7), and the preliminary calculation of clock frequencies. The global time offset of each camera was written into offset.txt.

**Figure:**
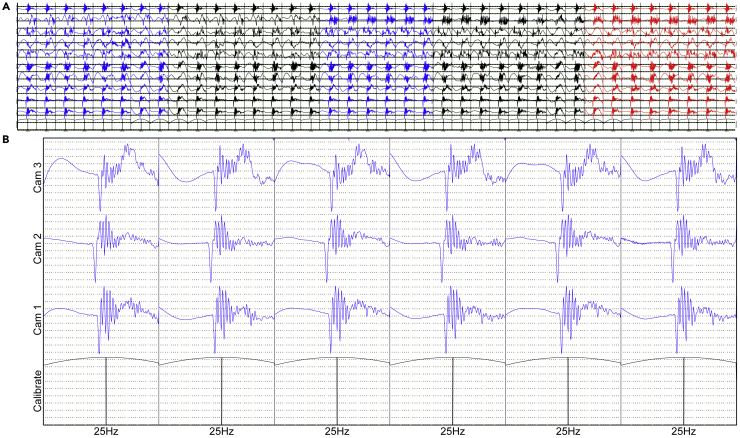
The waveforms of the received synchronization signals (A) Waveforms of ten cameras around the high-frequency sine waves for Step1(). (B) A partial magnification of 25Hz waveforms; the top three rows show waveform received by cameras #1-#3, and the bottom row shows the original waveform.

**Run****MATLAB****function step1.5()**, identifies all synchronization signals, and plots waveforms around the high-frequency sine waves. The time offsets for each 5-min interval from each camera were written into offsetb.txt.

**Run****MATLAB****function step2()**, calculates the clock frequency for each 5-min interval for each camera and resamples the sound data to let the clock frequency be equal among cameras. To let the combined frame shows the frames at the same time, the number of padding frames for each video were calculated and written into vpad.txt. Aligned and resampled audios were combined into a 20-channel WAV file. This file was then denoised by goldwave v5.5.8 to prevent the noises activating the sound source location.

**Run****MATLAB****function step3()**: this function locates the sound sources and generates some AAS subtitle files.

**Combine multi-view video**: the videos of cameras #1 to #9 were combined into a 4K, 30FPS multi-view video file by filmora v9.0. The videos are then inserted into different frames according to the number of padding frames. The arrangements of videos are as follows:

Top row: #9 #4 #5
Middle row: #3 #1 #2
Bottom row: #8 #7 #6

The frames of cameras #3, #1 and #2 are mirrored. The original videos are 2.7K with an aspect ratio of 4:3. These were clipped into 16:9 frames and a wide-angle distortion correction was applied. The audio stream of camera #1 was used and the other audio streams were deleted.

#### The cut-off of response alarm calls

Assuming the non-response alarm calls are independently uttered by individual monkeys, then the occurrence of alarm calls during a feeding bout is a non-homogeneous Poisson process. Because the intensity is not greatly changed within a small interval (e.g., < 30 s), we can use a homogeneous Poisson process to approximate the non-homogeneous Poisson process. In this case, the interval of a non-response alarm call to a previous alarm call is in accordance with an exponential distribution, and whose probability density function (PDF) is shown by the black curve in Figure 8A.

Similarly, the frequency of response alarm calls is initially high but decreases over time. We therefore also use an exponential distribution to approximate the distribution of the interval of response alarm calls. The PDF of intervals of response alarm calls is shown by the gray curve in Figure 8A. The histogram of intervals of all alarms calls is shown in Figure 8B.

**Figure:**
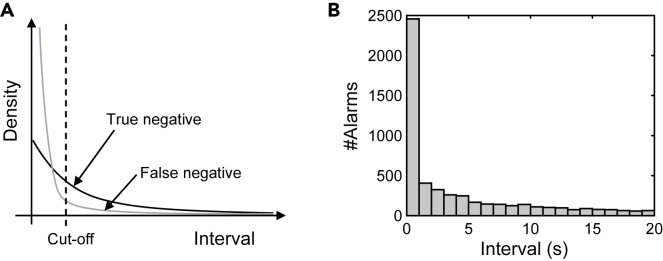
Fitting the alarm call intervals by two exponential distributions (A) The probability density function of intervals of response (gray) and non-response (black) alarm calls; (B) the histogram of intervals of alarm calls.

Figure 8A shows that regardless of the cut-off value, there are false positives (non-response alarm calls are misclassified as responses) and false negatives (response alarm calls been misclassified as non-responses). If the cut-off is too small, then there would be many false negatives, which reduces the accuracy of the results (increased bias); if the cut-off is too large then many useful data are discarded, which reduces the precision of our results (increased variance). The optimal cut-off can simultaneously retain non-response alarm calls and exclude response alarm calls, and the negative predictive value (NPV) can be used to evaluate the cut-off, which is calculated by

$$NPV=\frac{TNR}{FNR+TNR}\text{.}$$

Where TNR and FNR denote the true negative rate and false negative rate, respectively. We think an NPV of 0.95 is acceptable and would not greatly bias the results.

During the same feeding bout, the intensity of alarms is a function of time. In different bouts, the intensity functions will differ. Therefore, the interval of response or non-response alarms in the whole dataset are a mixture of multiple exponential distributions, whose PDF can be described by

$$f\left(x\right)=\int_{0}^{\infty }f_{\lambda }\left(u\right)f\left(x|\lambda =u\right)du=\int_{0}^{\infty }f_{\lambda }\left(u\right)ue^{-ux}du\text{.}$$

Where $f_{\lambda }\left(u\right)$ is the PDF of the intensity λ.

If $f_{\lambda }\left(u\right)$ can be assumed equal to a constant λ in all conditions (say non-admixed model), then the above equation is reduced to an exponential distribution, whose PDF is

$$f\left(x\right)=\lambda e^{-\lambda x}\text{.}$$

Otherwise (say admixed model), we use a Gamma distribution to approximate the distribution of λ because: (i) the support of a Gamma distribution is semi-infinite and is subject to the constraint λ>0; (ii) the PDF of a Gamma distribution is bell-curved in some conditions; (iii) it is easy to calculate the integral $\int_{0}^{\infty }f_{\lambda }\left(u\right)ue^{-ux}du$. We obtain the PDF of the interval under an admixed model:

$$f\left(x\right)=\int_{0}^{\infty }\frac{\beta^{\alpha }u^{\alpha -1}e^{-\beta u}}{\Gamma \left(\alpha \right)}ue^{-ux}du=\frac{\alpha \beta^{\alpha }}{{\left(\beta +x\right)}^{1+\alpha }}\text{.}$$

We use an additional parameter q to describe the proportion of response alarm calls, and establish four candidate models, with the intervals of response or non-response alarms approximated by either mixed or non-mixed models. We use a down-hill simplex algorithm to estimate the parameters and select the model with the optimal BIC (File S10). The parameters, likelihoods, BICs and estimates of parameters of these four models are given in Table S13.

Model 2, that uses a non-mixed model for the response alarms and a mixed model for the non-response alarms, has the lowest BIC and is thus selected. The fitted PDFs of interval of response and non-response alarm calls and the NPV as a function of cut-off values are shown in Figures 9 and 10, respectively. In model 2, the cut-off of 5.5 s (equivalent to 5 s for rounded time in seconds) has an NPV of 0.9656 and is used in this study.

**Figure:**
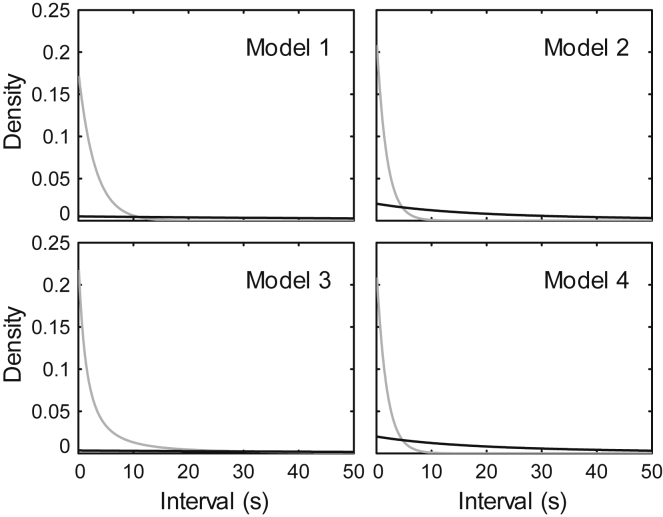
The fitted probability density functions of intervals of response (gray) and non-response (black) alarm calls of four candidate models

**Figure:**
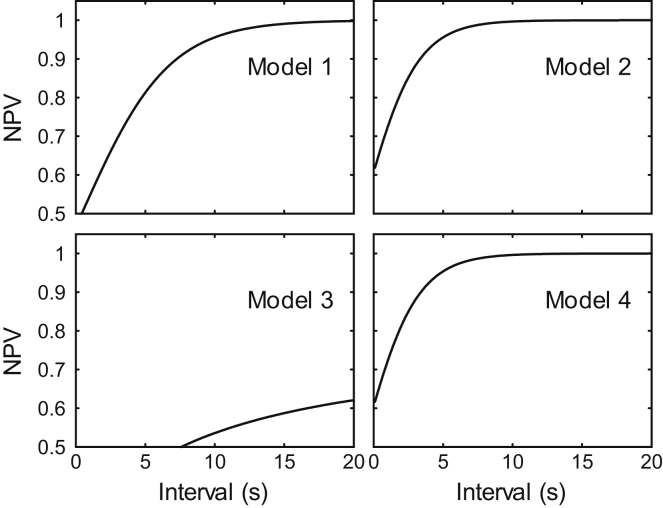
The negative predictive value as a function of cut-off values of four candidate models

#### Descriptive statistics

The raw data (File S1) were analyzed by a C# program (File S2) to generate spread sheets for subsequent analyses (Files S4-7). A feeding case is defined at the OMU level, which begins at the beginning of the feeding bout and ends when the last member of the OMU left the feeding area. We used a histogram to show the distribution of times of alarm calls in each feeding case, and are min-max scaled to the range of [0,1] (File S6). The rate of alarm calls of a feeding case is calculated by N/(M·E), where N is the number of alarm calls uttered by members of the focal OMU, M is the number of individuals (excluding infants) of the OMU and E is the duration of the feeding case. The average rate of alarm calls was estimated as $\sum_{i}N_{i}/\sum_{i}M_{i}E_{i}$ with the SE estimated as: $\hat{SE}=\sqrt{\sum_{i}N_{i}/{\left(\sum_{i}M_{i}E_{i}\right)}^{2}}\text{.}$ We used a violin plot to show the distribution of rates of alarm calls in different seasons (File S4).

#### OMU ranking

The dominance proportions between OMUs were calculated from the data of agonistic interactions for each interval, following the method of Guo et al.,^33^ which are used to calculated the David’s Score of each OMU.^49^^,^^50^ The ranks of OMUs were converted from the David’s Score (File S2).

#### RRDA rate

We used the *Poisson generalized linear mixed model* (Poisson GLMM) to measure independently the influence of several factors associated with RRDA. The response variable Y is the number of alarms an individual made during a feeding case (discrete) and is assumed to accord with a Poisson distribution, the exposure E is the duration of the feeding case (continuous). The following explanatory variables matrix X are included: season (categorical), time of day (binary: 1 for morning and 2 for afternoon), temperature (continuous, in Celsius degree), weather (binary: one for clear and cloudy weather, and zero for in windy, foggy and rainy weather), number of OMUs present (in the feeding bout, discrete), average rank of OMUs present (continuous), average feeding time across OMUs (continuous, from enter to leave), age-sex class (categorial), OMU rank (discrete), OMU size (discrete) and the identity of the caller (categorial, as a random factor to avoid pseudo-replication).

The former seven factors (season, time of day, temperature, weather, number of OMUs presented, average rank of OMUs presented, average feeding time across OMUs) reflect the intensity of food competition (Prediction 1), e.g., the GSMs required more energy than could be obtained from foraging in winter,^29^ and they have higher heat loss in windy, foggy or rainy weather, or in low temperature, when more OMUs feed together they feed quicker; the latter three factors (age-sex class, OMU rank and OMU size) are used to study the pattern of the deceptive alarm calls in the GSM (Prediction 2). The *generalized variance inflation factor* (GVIF) was used to evaluate multi-collinearity in the explanatory variables, with variables with a $\sqrt[{df}]{GVIF}<5$ excluded.^51^

The Poisson GLMM was performed using the glmer function^52^ in r (File S5). The effects of factors were tested by analysis of deviance using the Anova function in the car package. To control the rate of type I errors, a *false discovery rate* (FDR) correction was applied the P values. The multiple comparison of means between seasons are performed by the Tukey’s method in emmeans package. The significance level α was set at 0.05.

#### Changes of RRDA rate over time

For a feeding bout, the food competitors are reduced over time, which may affect the degree of food competition and the RRDA rate. To explore whether the RRDA rate changes over time, we consider the RRDAs in a feeding case as a non-homogeneous Poisson point process and the RRDA rate as its intensity, and developed a Poisson intensity regression. The intensity function of this random process is:

$$\lambda \left(X,t\right)=M\;\exp\left(X\beta +t\beta_{t}\right)\text{,}$$

where M is the number of individuals within the OMU, t is the elapsed time from the beginning of the feeding case, $\beta_{t}$ is the regression coefficient of the elapsed time, and the covariates X are those that may influence the intensity of the Poisson process, consisting of season (categorical), time of day (binary), weather (binary), number of OMUs present (discrete), average rank of OMUs present (continuous), average feeding time across OMUs (continuous), OMU rank (discrete), OMU size (discrete), and the identity of OMU (categorial). The null hypothesis is $\beta_{t}=0$.

Let $D_{i}$ be the number of RRDAs observed and $t_{ij}$ be the time of the *j*^th^ RRDA of feeding case i. The expected number of RRDAs in feeding case i is given by

$$\lambda_{i}=\int_{0}^{E_{i}}\lambda \left(X_{i},t\right)dt=\frac{1}{\beta_{t}}M_{i}\;\exp\left(X_{i}\beta \right)\left[exp\left(E_{i}\beta_{t}\right)-1\right]\text{.}$$

The natural logarithm of the likelihood of all feeding cases is

$$\ln\;L\left(D|\beta ,\beta_{t}\right)=-\sum_{i}\left(\lambda_{i}+D_{i}X_{i}\beta +D_{i}\;\ln\;M_{i}\right)+\beta_{t}\sum_{ij}t_{ij}\text{.}$$

The regression coefficients β and $\beta_{t}$ can be solved by the maximizing the likelihood. We used analysis of deviance to test the significance of $\beta_{t}$. The difference of deviance between the model considering time and the null model is

$$\Delta Deviance=2\;\ln\;L\left(D|\hat{\beta },{\hat{\beta }}_{t}\right)-2\;\ln\;L\left(D|\hat{\beta },0\right)\text{,}$$

which is asymptotically in accordance with a Chi-squared null distribution with a single degree of freedom. The right-tailed probability is used to test the null hypothesis. We also used a Wilcoxon’s sign-rank test to compare the median of number of RRDAs between the first and the second halves of each feeding case. Both analyses were performed by a MATLAB program (File S6).

#### Escape and response rates

The escape rate is the proportion of alarm calls that result in any receivers to escape (climb up a tree or flee from the feeding site), and response rate is the proportion of alarm calls that result in any response (including escapes and alarms). We use *logistic generalized linear mixed model* (logistic GLMM) to analyze the influencing factors of escape and response rates, respectively. The binary response variable Y is the escape or response result of an RRDA, which is equal to 1 when any receivers escape or response and 0 otherwise. The explanatory variables X consist of: facing direction (of the caller, binary: facing toward or backwards the feeding site), position (of the caller, binary: inside or outside the feeding site), season (categorial), time of day (binary), weather (binary), duration (of the feeding bout, continuous), average rank of OMUs present (continuous), number of OMUs present (discrete), time interval (to previous RRDA, continuous), elapsed time (from the beginning of feeding bout, continuous), current number of OMUs in the feeding site (discrete), OMU rank (of the caller, discrete), age-sex class (of the caller, categorial) and the identity of the caller (categorial, as a random factor to avoid pseudo-replication).

The first eleven variables may reflect the reliability of an alarm call, because (i) that predators usually come from the outside the feeding area so alarms with their callers facing away from the feeding site or outside the feeding site may be more reliable; (ii) if Prediction 1 holds, alarm calls made when food competition is high are less reliable. OMU rank and age-sex class are used to test whether GSMs are more likely to response to more reliable alarm callers.

The logistic GLMM was also performed using the glmer function (File S7). The test of coefficient and mean comparisons were performed by the same method as above.

### Quantification and statistical analysis

All results were repeatedly performed and confirmed by Supplemental File Sets. Quantification and statistical analysis are presented in method details.

## Data Availability

Data have been deposited at Mendeley Data and are publicly available as of the date of publication. DOIs are listed in the key resources table. All original code has been deposited at Mendeley Data and is publicly available as of the date of publication. DOIs are listed in the key resources table. Any additional information required to reanalyze the data reported in this paper is available from the lead contact upon request. The files in Mendeley Data are. File S1. Total.xlsx, the raw dataset including: alarm call records, OMU enter and leave records, agonistic interactions, initial family structure, individual dispersal records and feeding bout information. File S2. AlarmData.exe, the program (with its C# source code) to analyze the raw dataset. File S3. Alarm.m, the MATLAB video processing program. File S4. App1.R, the R code to analyze the distribution of RRDA rates in different seasons (violin plot). File S5. App2.R, the R code to analyze the influencing factors of RRDA rate by Poisson GLMM. File S6. App3.m, the MATLAB code to test whether RRDA intensity is a function of time by Poisson intensity regression. File S7. App4.R, the R code to analyze the influencing factors of RRDA escape and response rates by Logistic GLMM. File S8. Food.mp4, a video of an adult female (MB) used the ‘chuck’ calls to gain foods recorded at 16:09 Dec 24th, 2020. File S9. FigS9.m, the MATLAB code to compare two alarm calls (plotting waveform, spectrogram and spectrum). File S10. Cutoff.m, the MATLAB program to choose the optimal model and cut-off for response alarms.
