# Supplementary material for: Do golden snub-nosed monkeys use deceptive alarm calls during competition for food?
Source: iScience. 2023 Feb 2;26(2):106098. doi: 10.1016/j.isci.2023.106098 (PMC9958509; doi:10.1016/j.isci.2023.106098)
Supplement: Document S1. Tables S1–S13 and Figure S1 [file mmc1.pdf]

iScience, Volume *26*

Supplemental information

Do golden snub-nosed monkeys use deceptive alarm calls during competition for food?

Tiantian Wang, Yuchen Kong, He Zhang, Yuhang Li, Rong Hou, Derek W. Dunn, Xiduo Hou, Kang Huang, and Baoguo Li

Supplementary Tables

Table S1. Dominance hierarchies in the first interval (Oct. 2020 to Jan. 2021), Related to Table 1 and Table 2.

OMU	Rank	W_1	W_2	L_1	L_2	DS	NDS
DD	1	5.395	12.772	0.605	0.797	16.765	5.395
YQ	2	5.332	12.203	0.668	0.543	16.324	5.332
DX	3	4.000	6.816	2.000	1.817	6.998	4.000
HD	4	2.632	3.054	3.368	4.892	−2.573	2.632
XZ	5	2.520	2.768	3.480	5.166	−3.357	2.520
XL	6	1.005	0.301	4.995	10.273	−13.962	1.005
XW	7	0.115	0.246	5.885	14.671	−20.196	0.115

Table S2. Dominance proportions between OMUs in the first interval, Related to Table 1 and Table 2.

	DD	YQ	DX	HD	XZ	XL	XW
DD	*	0.600	0.880	0.944	0.971	1.000	1.000
YQ	0.400	*	0.963	0.985	0.984	1.000	1.000
DX	0.120	0.037	*	0.907	0.979	1.000	0.957
HD	0.056	0.015	0.093	*	0.469	1.000	1.000
XZ	0.029	0.016	0.021	0.531	*	0.923	1.000
XL	0.000	0.000	0.000	0.000	0.077	*	0.929
XW	0.000	0.000	0.043	0.000	0.000	0.071	*

Table S3. Dominance hierarchies in the second interval (Mar. 2021 to Jul. 2021), Related to Table 1 and Table 2.

OMU	Rank	W_1	W_2	L_1	L_2	DS	NDS
YQ	1	5.225	12.331	0.775	1.205	15.577	5.225
DD	2	5.076	12.170	0.924	1.791	14.531	5.076
XZ	3	3.692	6.690	2.308	3.231	4.843	3.692
DX	4	3.487	5.977	2.513	3.542	3.409	3.487
HD	5	2.276	2.228	3.724	5.846	−5.065	2.276
XL	6	0.789	1.378	5.211	12.433	−15.477	0.789
SZ	7	0.455	0.359	5.545	13.086	−17.818	0.455

Table S4. Dominance proportions between OMUs in the second interval, Related to Table 1 and Table 2.

	YQ	DD	XZ	DX	HD	XL	SZ
YQ	*	0.556	0.824	0.846	1.000	1.000	1.000
DD	0.444	*	0.923	0.875	1.000	0.833	1.000
XZ	0.176	0.077	*	0.615	0.900	0.923	1.000
DX	0.154	0.125	0.385	*	0.824	1.000	1.000
HD	0.000	0.000	0.100	0.176	*	1.000	1.000
XL	0.000	0.167	0.077	0.000	0.000	*	0.545
SZ	0.000	0.000	0.000	0.000	0.000	0.455	*

Table S5. Test for multi-collinearity in the Poisson regression, Related to Table 1.

Factor	GVIF	d.f.	$\sqrt[2df]{\text{GVIF}}$
Season	10.492	3	1.480
TimeOfDay	1.239	1	1.113
#OMUs	4.329	1	2.081
Weather	1.312	1	1.145
AvgFeedTime	1.615	1	1.271
AvgRank	4.556	1	2.134
Temperature	5.998	1	**2.449**
AgeSex	1.026	4	1.003
OMURank	1.289	1	1.135
OMUSize	1.606	1	1.267

Table S6. Effects of explanatory variables on the frequency of RRDAs, Related to Table 1.

Factor	β	SE	Z	P	Sig
(Intercept)	−8.991	0.298	−30.136	<0.001	***
Season2	−0.071	0.059	−1.192	0.233	
Season3	0.135	0.055	2.429	0.015	*
Season4	0.533	0.049	10.880	<0.001	***
TimeOfDay2	−0.099	0.033	−3.040	0.002	**
#OMUs	−0.139	0.032	−4.281	0.000	***
Weather2	0.086	0.035	2.477	0.013	*
AvgFeedTime	0.000	0.000	−5.579	0.000	***
AvgRank	0.396	0.065	6.098	0.000	***
AgeSexAM	−5.233	0.630	−8.311	<0.001	***
AgeSexJ	−0.332	0.177	−1.876	0.061	
AgeSexSF	0.472	0.399	1.181	0.238	
AgeSexSM	−1.083	0.236	−4.587	0.000	***
OMURank	0.044	0.024	1.802	0.072	
OMUSize	0.016	0.019	0.867	0.386	

Table S7. Inter-seasonal comparison of frequency of RRDAs, Related to Table 1.

Season pair	β	SE	Z	P	Sig
Spring−Summer	0.071	0.059	1.192	0.632	
Spring−Autumn	−0.135	0.055	−2.429	0.072	
Spring−Winter	−0.533	0.049	−10.880	<0.001	*******
Summer−Autumn	−0.205	0.060	−3.418	0.004	*****
Summer−Winter	−0.604	0.061	−9.934	<0.001	*******
Autumn−Winter	−0.399	0.048	−8.261	<0.001	*******

Table S8. Inter age−sex class comparison of frequency of RRDAs, Related to Table 1.

Class pair	β	SE	Z	P
AF−AM	5.233	0.630	8.311	**<0.001**
AF−J	0.332	0.177	1.876	0.330
AF−SF	−0.472	0.399	−1.181	0.762
AF−SM	1.083	0.236	4.587	**<0.001**
AM−J	−4.900	0.635	−7.719	**<0.001**
AM−SF	−5.704	0.730	−7.818	**<0.001**
AM−SM	−4.150	0.654	−6.347	**<0.001**
J−SF	−0.804	0.397	−2.027	0.253
J−SM	0.750	0.232	3.234	**0.011**
SF−SM	1.554	0.433	3.592	**0.003**

Table S9. Test for multi-collinearity in the two logistic regressions, Related to Table 2.

Factor	Escape rate			Response rate		
	GVIF	d.f.	$\sqrt[2df]{GVIF}$	GVIF	d.f.	$\sqrt[2df]{GVIF}$
Direction	1.060	1	1.030	1.059	1	1.029
Position	1.154	1	1.074	1.123	1	1.060
Season	2.114	3	1.133	2.095	3	1.131
TimeOfDay	1.045	1	1.022	1.039	1	1.019
Weather	1.197	1	1.094	1.179	1	1.086
Duration	1.587	1	1.260	1.516	1	1.231
#OMUs	5.723	1	**2.392**	5.148	1	**2.269**
AvgRank	5.398	1	**2.323**	4.892	1	2.212
Interval	1.232	1	1.110	1.185	1	1.088
Time	1.364	1	1.168	1.199	1	1.095
Current#OMUs	1.316	1	1.147	1.272	1	1.128
OMURank	1.197	1	1.094	1.158	1	1.076
AgeSex	1.081	4	1.010	1.064	4	1.008

Table S10. Effects of explanatory variables on escape rate, Related to Table 2.

Factor	β	SE	Z	P	Sig
(Intercept)	−2.683	0.388	−6.922	0.000	***

Direction2	−0.055	0.198	−0.276	0.783	
Position2	0.278	0.137	2.034	0.042	*
Season2	0.344	0.214	1.604	0.109	
Season3	0.528	0.204	2.584	0.010	**
Season4	0.512	0.183	2.803	0.005	**
TimeOfDay2	0.037	0.117	0.316	0.752	
Weather2	−0.045	0.126	−0.356	0.722	
Duration	0.000	0.000	−1.165	0.244	
Interval	0.001	0.000	3.945	0.000	***
Elapse	0.000	0.000	0.890	0.373	
Current#OMUs	0.025	0.050	0.498	0.619	
OMURank	−0.031	0.040	−0.764	0.445	
AgeSexAM	−11.170	256.000	−0.044	0.965	
AgeSexJ	−0.302	0.169	−1.787	0.074	.
AgeSexSF	−0.727	0.415	−1.751	0.080	.
AgeSexSM	0.410	0.255	1.609	0.108	

Table S11. Effects of explanatory variables on response rate, Related to Table 2.

Factor	β	SE	Z	P	Sig
(Intercept)	−1.358	0.313	−4.339	0.000	***
Direction2	0.182	0.123	1.485	0.137	
Position2	0.013	0.083	0.152	0.879	
Season2	0.090	0.120	0.748	0.454	
Season3	0.183	0.122	1.496	0.135	
Season4	0.508	0.102	4.967	0.000	***
TimeOfDay2	−0.084	0.070	−1.193	0.233	
Weather2	0.184	0.075	2.459	0.014	*
Duration	0.000	0.000	−0.644	0.519	
AvgRank	0.223	0.074	3.029	0.002	**
Interval	0.001	0.000	1.790	0.073	
Elapse	−0.001	0.000	−7.998	0.000	***
Current#OMUs	0.011	0.030	0.360	0.719	
OMURank	−0.017	0.025	−0.696	0.487	
AgeSexAM	−12.460	128.000	−0.097	0.922	
AgeSexJ	−0.323	0.104	−3.121	0.002	**
AgeSexSF	−0.405	0.231	−1.756	0.079	
AgeSexSM	−0.082	0.178	−0.463	0.643	

Table S12. Inter−seasonal variability comparison of response rate, Related to Table 2.

Season pair	β	SE	Z	P	Sig
Spring−Summer	−0.090	0.120	−0.748	0.877	
Spring−Autumn	−0.183	0.122	−1.496	0.440	
Spring−Winter	−0.508	0.102	−4.967	<0.001	***
Summer−Autumn	−0.093	0.123	−0.755	0.875	
Summer−Winter	−0.418	0.113	−3.695	0.001	**
Autumn−Winter	−0.325	0.106	−3.064	0.012	*

Table S13. The parameters, likelihoods, BICs and estimates of parameters of four candidate models, Related to STAR Methods.

Model	Parameters	Likelihood	BIC	Estimates of parameters
1	$\lambda_1,\ \lambda_2,\ q$	−32738.8	65504.4	0.333, 0.012, 0.535
2	$\lambda_1,\ \alpha_2,\ \beta_2,\ q$	−32131.5	64298.9*	0.560, 1.635, 49.180, 0.394
3	$\alpha_1,\ \beta_1,\ \lambda_2,\ q$	−32278.8	64593.5	0.820, 2.589, 0.013, 0.736
4	$\alpha_1,\ \beta_1,\ \alpha_2,\ \beta_2,\ q$	−32132.4	64309.7	129.425, 231.954, 1.641, 49.667, 0.395

The parameters with a subscript ‘1’ and ‘2’ are used for response and non-response alarm calls, respectively.

Supplementary Figures

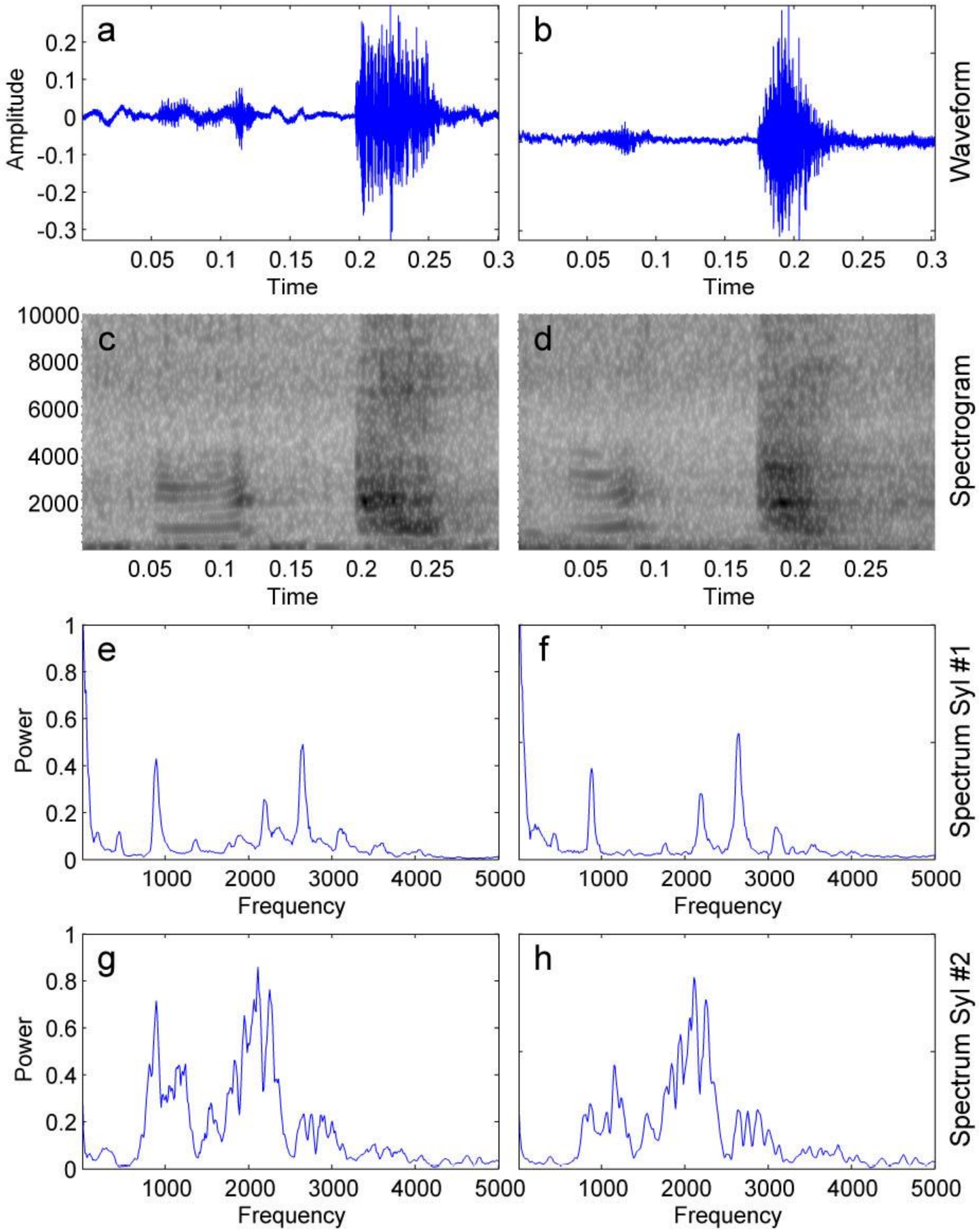

Figure S1. Comparisons of two alarm calls sent by adult males. Left: an environmental disturbance-elicited alarm (sent by YQ on 10:23:44 am, 9th Jun. 2021); right: a deceptive alarm (sent by XZ on 10:12:50 am, 29th Dec. 2021). (a) and (b): Waveform; (c) and (d): Spectrogram; (e) and (f): Spectrum of the first syllable. (g) and (h): Spectrum of the second syllable. Related to Table 2.
